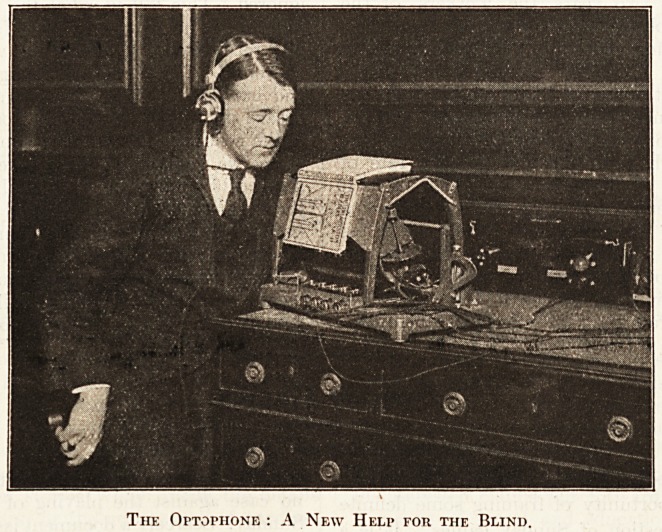# An Invention to Convey the Meaning of Ordinary Print

**Published:** 1921-02-26

**Authors:** 


### HOW THE BLIND MAY READ.

### An Invention to Convey the Meaning of Ordinary Print.

Below we publish an illustration of an instrument invented by Dr. E. E. Fournier d'Albe of London, and with which it is possible for a totally blind person to read ordinary printed matter. In a short note it is impossible to enter into the exact mechanism of this "Optophone," but, as its name implies, the principle consists in transmitting each printed letter to the reader's mind as a single musical note or a series of them. The actual medium of transmission, as can be seen in the photograph, is the usual telephone wire and receiver, and the printed matter, in this instance a book, is placed face downwards on the instrument, the recording mechanism passing over each letter in turn and producing the series sounds appropriate to the spelled-out word.

The two best-known systems of reading by the blind are "Braille" and "Moon." The former comprises combinations of embossed "dots" to stand for letters and words, and the latter is based on a number of complete signs not unlike Roman characters. To a certain extent both systems have disadvantages, despite the enormous comfort and entertainment they have afforded the blind. The books are necessarily specially printed, expensive, and bulky. Also it is stated that there is often with the old system considerable difficulty, especially for the adult blind, in acquiring the necessary delicacy of touch. With this new instrument it is claimed that both these disadvantages are overcome, although we may subscribe the opinion that the "Optophone" at its present price of £105 puts it beyond the reach of any but wealthy people or institutions, and also that, speaking of blind persons in general, it is doubtful whether their appreciation of pitch and variations in musical notes will be on the average more acute than their sense of touch. Undoubtedly both faculties are, or finally come to be, considerably refined, but the greater advantage of the new machine appears to be in the fact that printed matter generally, with the minimum of preparation, can be placed on the recorder and the words at once transmitted to the blind man's mind.

In any case, the instrument's mechanism and its use among those afflicted with the cruellest of disabilities are based upon the best scientific principles, and, as can be seen, it is neither so cumbersome nor so complicated that it cannot be of the greatest home or institutional use. We can readily believe the claim that, for a person who had learnt to read before sight was lost, a reasonable facility in use of the new instrument can be gained after comparatively few lessons.

The Optophone: A New Help for the Blind.

The following letter from the inventor makes it clear that too much must not be expected of this ingenious, but rather intricate and expensive, instrument.

"Sir,—In some of the reports lately published in the press concerning the recent optophone demonstrations at South Kensington, I see it stated that the method of 'reading by ear' is claimed to supersede the Braille method of reading by touch.

"It should be noted that no such claim is made by either the inventor or the manufacturers of the new instrument, nor would there be in any excuse such advancing a claim. The optophone is an expensive instrument which but few among the blind will be able to afford. It is rather more elaborate than a typewriter and less portable. Above all, it is quite useless to a person with defective hearing, such as is very prevalent among the blind.

"The National Institute for the Blind, as the pioneer Institute for the education of the blind through the sense of touch, is bound to continue its policy of extending the embossed literature which has already attained such great dimensions. At the same time it welcomes every genuine invention for the blind, and encourages it if it shows any promise. At the present moment an experimental class in optophone reading is being conducted on its premises in Great Portland Street, and the blind may rely upon the National Institute for the Blind to utilise the invention in their interests to the fullest extent warranted by the results obtained.

"E. E. Fournier d'Albe."

In addition, it must be pointed out that Braille is a system which can be written by a special typewriter or with a board and stylus, and in this respect could not be superseded by the optophone.

---

Mr. Ernest Pemberton-Barnes, of Battle, has left £150 each to St. Dunstan's, British Home for Incurables, Streatham, and the Cancer Hospital.

## Figures and Tables

**Figure f1:**